# Supplementary material for: Proenkephalin-A secreted by renal proximal tubules functions as a brake in kidney regeneration
Source: Nat Commun. 2023 Nov 7;14:7167. doi: 10.1038/s41467-023-42929-5 (PMC10630464; doi:10.1038/s41467-023-42929-5)
Supplement: Supplementary file 1 — Supplementary Information [file 41467_2023_42929_MOESM1_ESM.pdf]

Supplementary Information

Proenkephalin-A Secreted by Renal Proximal Tubules Functions as a Brake in Kidney Regeneration

Chi Liu et al.

Supplementary Figures 1–5

Supplementary Tables S1-S2

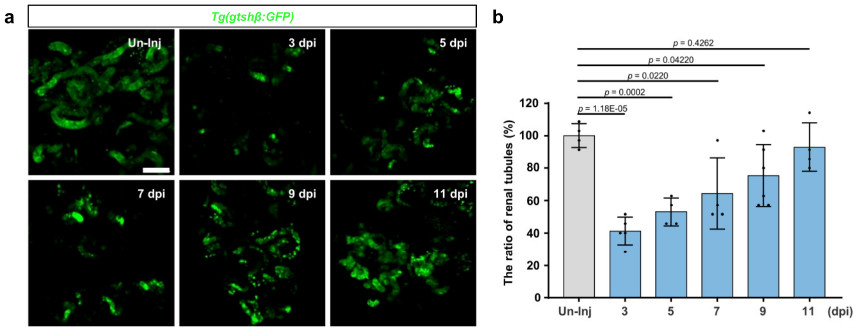

Fig. S1 Changes in PTs after AKI in zebrafish kidneys.

a Confocal images showing adult *Tg(gtshβ:GFP)* kidneys after AKI. Scale bar, 50 μm. **b** The relative number the number of *gtshβ:GFP*-labeled PTs per kidney (n = 4 in 3 dpi group, n = 6 in 9 dpi group, and n = 4 in other groups) was quantified for each condition in a. The data are presented as the fold change relative to the Un-Inj group (set as 100%). Data were analyzed by two-sided t-test and are presented as mean values ± SD.

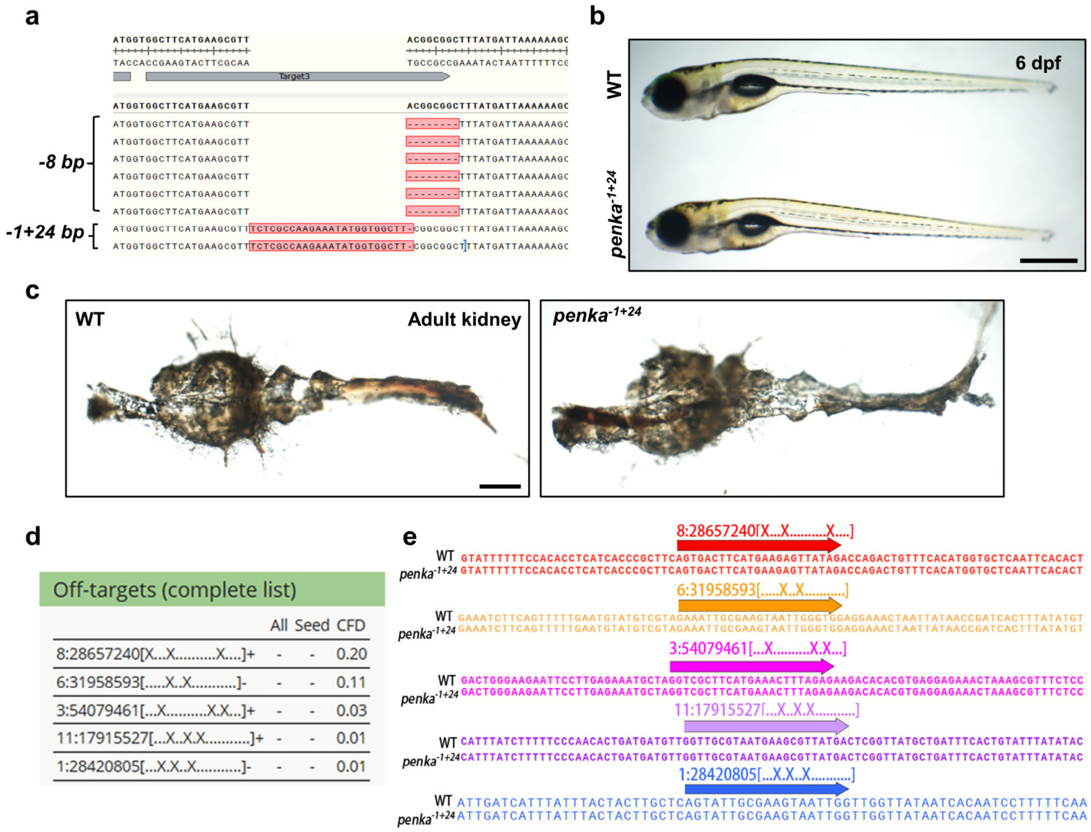

Fig. S2 Generation of *penka* mutants using CRISPR/Cas9 technology.

a Alignment of genomic sequences of WT, *penka*$^{-1+24}$ and *penka*$^{-8}$ zebrafish. **b** Embryo morphology of WT and *penka*$^{-1+24}$ zebrafish at 6 dpf. *penka*$^{-1+24}$ embryos were morphologically indistinguishable from WT embryos. **c** Kidneys of adult WT and *penka*$^{-1+24}$ zebrafish. There were no differences between WT and *penka*$^{-1+24}$ zebrafish, including in the size and structure of the kidneys (n = 9). **d** The top five putative off-target sites for the target CRISPR sequence of the zebrafish *penka*$^{-1+24}$ mutant were predicted using CRISPRScan. **e** Sanger sequencing of putative off-target sites revealed no detectable indel formation at these locations. Scale bars in b and d, 600 μm.

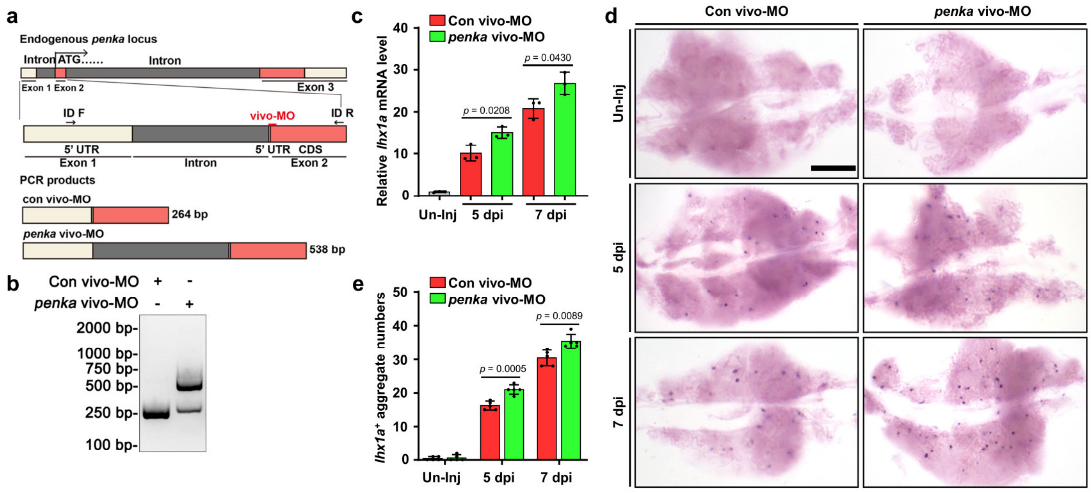

Fig. S3 Knockdown of *penka* promotes kidney regeneration.

a Schematic of the *penka* gene and the strategy for identifying the effect of *penka* vivo-MO. The *penka* gene consists of 3 exons and 2 introns. We designed an antisense *penka* vivo-MO against the donor splice site of intron-exon 2 (red line) of the zebrafish *penka* gene, which can block the splicing of this intron. The identification forward primer (ID F) and reverse primer (ID R) are indicated as arrows, and the size of the RT-PCR products was predicted. **b** RT-PCR analysis of *penka* in 7 dpi kidneys after administration (at 2, 4 and 6 dpi) of *penka* vivo-MO or Con vivo-MO following AKI. The primers ID F and ID R were utilized to detect splicing variants. **c, d** qRT-PCR (**c**) and WISH (**d**) analyses of *lhx1a* after administration of *penka* vivo-MO or Con vivo-MO following AKI (n = 3). The data in c are presented as the fold change the relative to the Un-Inj groups. **e** Quantitation of *lhx1a*$^{+}$ RPCAs per kidney (n = 5) was performed for each condition in d. The data in c and e were analyzed by two-sided t-test and are presented as mean values ± SD. Scale bar in d, 600 µm.

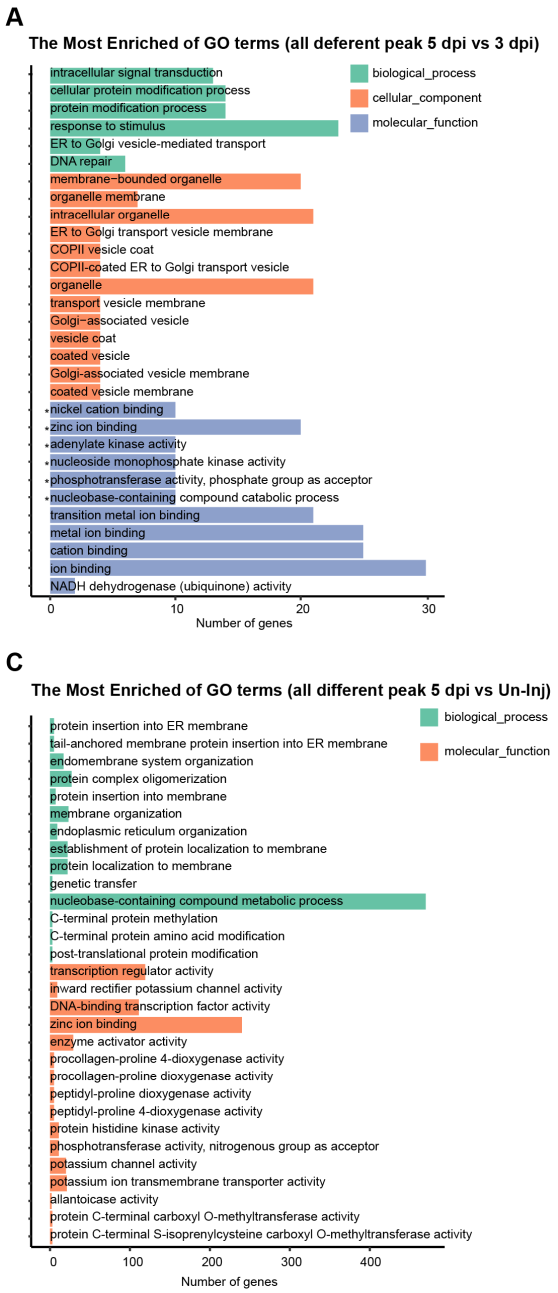

Fig. S4 GO enrichment analysis after ChIP-seq.

a-c GO enrichment analysis of signaling in 5 dpi kidneys compared with 3 dpi kidneys (**a**), 3 dpi kidneys compared with Un-Inj kidneys (**b**), and 5 dpi kidneys compared with Un-Inj kidneys (**c**). The data were analyzed by analysis of variance (ANOVA); $*p < 0.05$.

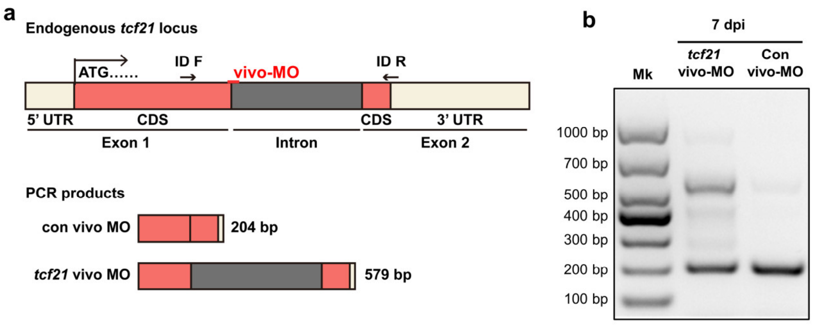

Fig. S5 Efficiency verification of *tcf21* vivo-MO.

a Schematic of the *tcf21* gene and the strategy for identification of the effect of *tcf21* vivo-MO. *tcf21* consists of 2 exons and 1 intron. To target the donor splice site of exon 1-intron (red line) in the zebrafish *tcf21* gene, we designed an antisense *tcf21* vivo-MO, which can block the splicing of this intron. The identification forward primer (ID F) and reverse primer (ID R) are depicted as arrows, and the predicted sizes of RT-PCR products are shown. **b** RT-PCR analysis of *tcf21* in 7 dpi kidneys after administration (at 2, 4 and 6 dpi) of *tcf21* vivo-MO or Con vivo-MO following AKI. The primers ID F and ID R were utilized to detect splicing variants.

Table S1, Oligonucleotides

Target gene	Name	Sequence 5’-3’
tcf21, zebrafish	*tcf21*-RT F	ggatgaatttcacgaatctgag
tcf21, zebrafish	*tcf21*-RT R	ggaagctgtagtcccgcataaac
tcf21, zebrafish	*tcf21*-qRT F	gcacctgcgacagatactcgccaatg
tcf21, zebrafish	*tcf21*-qRT R	gcatttctttgagttcattttctgg
tcf21, zebrafish	*tcf21*-probe F	gtctccagccaacatgtccac
tcf21, zebrafish	*tcf21*-probe R	gtccatcttgagtctcaggaag
tcf21, zebrafish	*tcf21* vivo-MO	gtgtctcaccaggttgacggatgt
tcf21, zebrafish	*tcf21* vivo-MO ID-F	ggacaccaaactctccaagctggac
tcf21, zebrafish	*tcf21* vivo-MO ID-R	tgagtctcaggaagctgtagtcccg
lhx1a, zebrafish	*lhx1a*-qRT F	gcaagagacgggactcaaca
lhx1a, zebrafish	*lhx1a*-qRT R	cacgagcgttcgcattcttc
penka, zebrafish	*penka*-RT F	gaactcctggtggactgtg
penka, zebrafish	*penka*-RT R	ctcctgtacactcctcatgaag
penka, zebrafish	*penka*-probe F	atggcgttaatgatgaactcctggtg
penka, zebrafish	*penka*-probe R	atccatgaatcctccgtatctcttctg
penka, zebrafish	*penka* vivo-MO	acagctcatggcgttaatgatgaac
penka, zebrafish	*penka* vivo-MO ID-F	cgtgataagcggctggctatagtgag
penka, zebrafish	*penka* vivo-MO ID-R	cagtgtgtcgatgtctgtctgtcg
penka, zebrafish	*penka* mutant ID-F	tgacagcattttccactgcg
penka, zebrafish	*penka* mutant ID-R	acagtgcaagctctacacaaa
slc20a1a, zebrafish	*slc20a1a*-probe F	catcggaggatcggcagaaacc
slc20a1a, zebrafish	*slc20a1a*-probe R	tccgatgttggaagcgacaacc
trpm7, zebrafish	*trpm7*-probe F	atgtcccagaagtcctggatcg
trpm7, zebrafish	*trpm7*-probe R	cgtatgccagtatgtccgcagc
PENK, human	*PENK*-probe F	atggcgcggttcctgacactttg
PENK, human	*PENK*-probe R	ccatcaacagtttcccactggagg

Table S2, Reagent and resource.

REAGENT or RESOURCE	SOURCE	IDENTIFIER
**Antibodies**		
Anti-H3K4 me3, rabbit monoclonal	CST, 9751S; Dilution, 1:500	RRID:AB_2616028
Anti-Histone H3, rabbit polyclonal	Abcam, ab1791; Dilution, 1:1000	RRID:AB_302613
Anti-Met Enkephalin, rabbit polyclonal	Abcam, ab22620; Dilution, 1:200	RRID:AB_447201
Anti-Pax2a, rabbit polyclonal	Abcam, ab229318; Dilution, 1:200	[25]
Anti-β-actin, mouse monoclonal	Beyotime，AA128; Dilution, 1:5000	RRID:AB_2861213
HRP-conjugated goat Anti-mouse IgG(H+L), goat polyclonal	Protein-tech, SA00001-1; Dilution, 1:5000	RRID:AB_2722565
HRP-conjugated goat anti-rabbit IgG, goat polyclonal	Protein-tech, SA00001-2; Dilution, 1: 5000	RRID:AB_2722564
Goat anti-rabbit IgG (H+L), Alexa fluor 633, goat polyclonal	Invitrogen, A21070; Dilution, 1:500	RRID:AB_2535731
Sheep anti-digoxigenin-peroxidase antibody，Sheep polyclonal	Roche, 11207733910; Dilution, 1:500	RRID:AB_514500
Sheep anti-digoxigenin-alkaline phosphatase antibody	Roche, 11093274910; Dilution, 1:2000	RRID:AB_514497
**Chemicals and Peptides**		
Tramadol	Grunenthal, J20050051	
Naloxone methylide	APExBIO, B8208	
VAS2870	Sigma, SML0273	
CPI-455	Selleck, S8287	
PBSF	Santa Cruz, SC-205429A	
LTL	Vector Laboratories, FL-1321-2	
Tyr-Gly-Gly-Phe-Met-OH (Met-ENK)	MedChemExpress, HY-P0073	
**Critical Commercial Assays**		
CUT&RUN Assay kit	CST, 86652	
Fluorimetric hydrogen peroxide assay kit	Sigma, MAK166	
Prime script II 1st strand cDNA synthesis Kit	Takara, 9767	
TB Green Premix EX Taq II	Takara, RR820A	
**Zebrafish lines**		
penka^{-1+24} mutant	This paper	
penka^{+8} mutant	This paper	
Tg(cdh17:DsRed)	[25, 52]	
Tg(hsp70l:tcf21)	This paper	
Tg(hsp70l:penka)	This paper	
Tg(gtshβ:GFP)	[45]	
Tg(lhx1a:DsRed)	[25]	
