# Supplementary material for: Proenkephalin-A secreted by renal proximal tubules functions as a brake in kidney regeneration
Source: Nat Commun. 2023 Nov 7;14:7167. doi: 10.1038/s41467-023-42929-5 (PMC10630464; doi:10.1038/s41467-023-42929-5)
Supplement: Supplementary file 3 — Reporting Summary [file 41467_2023_42929_MOESM3_ESM.pdf]

nature portfolio

Corresponding author(s): Chi Liu

Last updated by author(s): Oct 16, 2023

Reporting Summary

Nature Portfolio wishes to improve the reproducibility of the work that we publish. This form provides structure for consistency and transparency in reporting. For further information on Nature Portfolio policies, see our Editorial Policies and the Editorial Policy Checklist.

Statistics

For all statistical analyses, confirm that the following items are present in the figure legend, table legend, main text, or Methods section.

n/a	Confirmed	
☐	☒	The exact sample size (*n*) for each experimental group/condition, given as a discrete number and unit of measurement
☐	☒	A statement on whether measurements were taken from distinct samples or whether the same sample was measured repeatedly
☐	☒	The statistical test(s) used AND whether they are one- or two-sided<br>*Only common tests should be described solely by name; describe more complex techniques in the Methods section.*
☒	☐	A description of all covariates tested
☒	☐	A description of any assumptions or corrections, such as tests of normality and adjustment for multiple comparisons
☐	☒	A full description of the statistical parameters including central tendency (e.g. means) or other basic estimates (e.g. regression coefficient) AND variation (e.g. standard deviation) or associated estimates of uncertainty (e.g. confidence intervals)
☐	☒	For null hypothesis testing, the test statistic (e.g. *F*, *t*, *r*) with confidence intervals, effect sizes, degrees of freedom and *P* value noted<br>*Give P values as exact values whenever suitable.*
☒	☐	For Bayesian analysis, information on the choice of priors and Markov chain Monte Carlo settings
☒	☐	For hierarchical and complex designs, identification of the appropriate level for tests and full reporting of outcomes
☒	☐	Estimates of effect sizes (e.g. Cohen's *d*, Pearson's *r*), indicating how they were calculated

Our web collection on statistics for biologists contains articles on many of the points above.

Software and code

Policy information about availability of computer code

Data collection	Excel (Microsoft Office Home and Student 2019 version), imagej (with 64-bit JAVA8).
Data analysis	Burrows–Wheeler Aligner (BWA, v0.7.12), BWA-MEM (v0.7.12), Graphpad Prism (version 8.02), Seurat package (version 4.2.0).

For manuscripts utilizing custom algorithms or software that are central to the research but not yet described in published literature, software must be made available to editors and reviewers. We strongly encourage code deposition in a community repository (e.g. GitHub). See the Nature Portfolio guidelines for submitting code & software for further information.

Data

Policy information about availability of data

All manuscripts must include a data availability statement. This statement should provide the following information, where applicable:

- Accession codes, unique identifiers, or web links for publicly available datasets
- A description of any restrictions on data availability
- For clinical datasets or third party data, please ensure that the statement adheres to our policy

All datasets generated in this study have been deposited in the Gene Expression Omnibus repository under the series number GSE217831 (https://www.ncbi.nlm.nih.gov/geo/query/acc.cgi?acc=GSE217831). Source data are provided with this paper.

Research involving human participants, their data, or biological material

Policy information about studies with human participants or human data. See also policy information about sex, gender (identity/presentation), and sexual orientation and race, ethnicity and racism.

Reporting on sex and gender	Renal biopsies were performed as part of routine clinical diagnostic investigations. All kidney samples in this study were collected from renal biopsies. There are 3 male and 3 female patients.
Reporting on race, ethnicity, or other socially relevant groupings	The studied was conducted in an homogenous group of participants of Chinese ancestry.
Population characteristics	All patients were between 18 and 55 years old. Three patients with AKI and three patients with no detectable lesions verified by renal biopsy were enrolled in this study from the Department of Nephrology, Xinqiao Hospital, Chongqing, China. Patients with inflammatory and autoimmune-associated diseases, diabetes, polycystic kidney disease, and pregnancy were excluded from the study. Kidney biopsies were obtained from these patients for FISH analysis.
Recruitment	Patients were recruited from the Department of Nephrology, Xinqiao Hospital, Chongqing, China. All the participants were randomly selected for our analysis.
Ethics oversight	The human studies conducted in this research were approved by the Ethics Committee of Xinqiao Hospital, Army Medical University. The study design and conduct adhered to all applicable regulations concerning the use of human participants and were in accordance with the principles set forth in the Declaration of Helsinki. Informed written consent was provided by all participants. Furthermore, the study complies with the guidelines provided by the Ministry of Science and Technology (MOST) for the Review and Approval of Human Genetic Resources.

Note that full information on the approval of the study protocol must also be provided in the manuscript.

Field-specific reporting

Please select the one below that is the best fit for your research. If you are not sure, read the appropriate sections before making your selection.

[x] Life sciences [ ] Behavioural & social sciences [ ] Ecological, evolutionary & environmental sciences

For a reference copy of the document with all sections, see nature.com/documents/nr-reporting-summary-flat.pdf

Life sciences study design

All studies must disclose on these points even when the disclosure is negative.

Sample size	The sample size was not predetermined by the statistical method, but based on our previous studies (eg. Liu et al., Elife 2023; Chen et al., BIOCHEMICAL AND BIOPHYSICAL RESEARCH COMMUNICATIONS 2019)
Data exclusions	No data were excluded from the analysis.
Replication	At least 3 independent replicates of all data were performed and the exact n-values are shown in the figure legends. All attempts at replication were successful.
Randomization	Sample and participants were randomly allocated into the experimental groups.
Blinding	The investigators were blinded to group allocation during data collection and/or analysis.

Reporting for specific materials, systems and methods

We require information from authors about some types of materials, experimental systems and methods used in many studies. Here, indicate whether each material, system or method listed is relevant to your study. If you are not sure if a list item applies to your research, read the appropriate section before selecting a response.

Materials & experimental systems

n/a	Involved in the study
☐	☒ Antibodies
☒	☐ Eukaryotic cell lines
☒	☐ Palaeontology and archaeology
☐	☒ Animals and other organisms
☒	☐ Clinical data
☒	☐ Dual use research of concern
☒	☐ Plants

Methods

n/a	Involved in the study
☐	☒ ChIP-seq
☒	☐ Flow cytometry
☒	☐ MRI-based neuroimaging

Antibodies

Antibodies used

Anti-H3K4 me3, rabbit monoclonal; 1:500; CST, 9751S; RRID:AB_2616028.
Anti-Histone H3, rabbit polyclonal; 1:1000; Abcam, ab1791 ; RRID:AB_302613.
Anti-Met Enkephalin, rabbit polyclonal ; 1:200; Abcam, ab22620; RRID:AB_447201.
Anti-Pax2a, rabbit polyclonal ;1:200; Abcam, ab229318 .
Anti-β-actin, mouse monoclonal; 1:5000; Beyotime, AA128 ; RRID:AB_2861213.
HRP-conjugated goat Anti-mouse IgG(H+L), goat polyclonal; 1:5000; Protein-tech, SA00001-1; RRID:AB_2722565.
HRP-conjugated goat anti-rabbit IgG, goat polyclonal ; 1:5000; Protein-tech, SA00001-2 ; RRID:AB_2722564.
Goat anti-rabbit IgG (H+L), Alexa fluor 633, goat polyclonal ; 1:500; Invitrogen, A21070 . RRID:AB_2535731.
Sheep anti-digoxigenin-peroxidase antibody, Sheep polyclonal; 1:500; Roche, 11207733910; RRID:AB_514500.
Sheep anti-digoxigenin-alkaline phosphatase antibody; 1:2000; Roche, 11093274910; RRID:AB_514497.

Validation

All antibodies have been validated for the species and application, and the validation statements can be found on the manufacturers' website or the references. We did not use non-commercial antibodies.
Anti-H3K4 me3, rabbit monoclonal: https://www.cellsignal.cn/products/primary-antibodies/tri-methyl-histone-h3-lys4-c42d8-rabbit-mab/9751?Ns=product.currentLot.numberOfApplications|1&N=4294955687+4294960088&Nrpp=60&fromPage=plp.
Anti-Histone H3, rabbit polyclonal: https://www.abcam.cn/products/primary-antibodies/histone-h3-antibody-nuclear-marker-and-chip-grade-ab1791.html.
Anti-Met Enkephalin, rabbit polyclonal: https://www.abcam.cn/products/primary-antibodies/met-enkephalin-antibody-ab22620.html
Anti-Pax2a, rabbit polyclonal: https://www.abcam.cn/products/primary-antibodies/pax2-antibody-ab229318.html
Anti-β-actin, mouse monoclonal: https://www.beyotime.com/mobilegoods.do?method=code&code=AA128.
HRP-conjugated goat Anti-mouse IgG(H+L), goat polyclonal: https://www.ptgcn.com/Products/-Peroxidase-conjugated-Affinipure-FC-Fragment-Specific-Goat-Anti-Mouse-IgG-H-L.htm.
HRP-conjugated goat anti-rabbit IgG, goat polyclonal: https://www.ptgcn.com/products/HRP-conjugated-Affinipure-Goat-Anti-Rabbit-IgG-H-L-secondary-antibody.htm
Goat anti-rabbit IgG (H+L), Alexa fluor 633, goat polyclonal: https://www.thermofisher.cn/cn/zh/antibody/product/Goat-anti-Rabbit-IgG-H-L-Cross-Adsorbed-Secondary-Antibody-Polyclonal/A-21070.
Sheep anti-digoxigenin-peroxidase antibody, Sheep polyclonal: https://www.sigmaaldrich.cn/CN/zh/product/roche/11207733910.
Sheep anti-digoxigenin-alkaline phosphatase antibody: https://www.sigmaaldrich.cn/CN/zh/product/roche/11093274910.

Animals and other research organisms

Policy information about studies involving animals; ARRIVE guidelines recommended for reporting animal research, and Sex and Gender in Research

Laboratory animals

he following transgenic lines were used in this study: Tg(lhx1a:DsRed), Tg(cdh17:DsRed), Tg(gtshβ:GFP), Tg(hsp70l:penka), and Tg (hsp70l:tcf21). The AB strain of zebrafish served as the WT control for this study.
Zebrafish were produced, grown, and maintained according to standard protocols (https://zfin.org/zf_info/zfbook/zfbk.html). Adult zebrafish aged between 3 to 12 months were used for the experiments, and approximately equal sex ratios were employed.

Wild animals

No wild animals were used in the study.

Reporting on sex

Adult zebrafish approximately equal sex ratios were employed in this study.

Field-collected samples

No field collected samples were used in this study.

Ethics oversight

Animal care and use protocol was approved by the Institutional Animal Care and Use Committee of the Army Medical University, China (SYXK-PLA-2007035).

Note that full information on the approval of the study protocol must also be provided in the manuscript.

Plants

Seed stocks

Report on the source of all seed stocks or other plant material used. If applicable, state the seed stock centre and catalogue number. If plant specimens were collected from the field, describe the collection location, date and sampling procedures.

Novel plant genotypes

Describe the methods by which all novel plant genotypes were produced. This includes those generated by transgenic approaches, gene editing, chemical/radiation-based mutagenesis and hybridization. For transgenic lines, describe the transformation method, the number of independent lines analyzed and the generation upon which experiments were performed. For gene-edited lines, describe the editor used, the endogenous sequence targeted for editing, the targeting guide RNA sequence (if applicable) and how the editor was applied.

Authentication

Describe any authentication procedures for each seed stock used or novel genotype generated. Describe any experiments used to assess the effect of a mutation and, where applicable, how potential secondary effects (e.g. second site T-DNA insertions, mosiacism, off-target gene editing) were examined.

ChIP-seq

Data deposition

☒ Confirm that both raw and final processed data have been deposited in a public database such as GEO.

☒ Confirm that you have deposited or provided access to graph files (e.g. BED files) for the called peaks.

Data access links
May remain private before publication.

https://www.ncbi.nlm.nih.gov/geo/query/acc.cgi?acc=GSE217831

Files in database submission

https://www.ncbi.nlm.nih.gov/geo/query/acc.cgi?acc=GSE217831

Genome browser session
(e.g. UCSC)

https://www.ncbi.nlm.nih.gov/geo/query/acc.cgi?acc=GSE217831

Methodology

Replicates

Total DNA from uninjured zebrafish kidneys and injured zebrafish kidneys at 3 dpi and 5 dpi was used for ChIP assays.

Sequencing depth

samples were sequenced on Illumina NovaSeq 6000, pair end.

Antibodies

Anti-H3K4 me3, rabbit monoclonal; CST, 9751S; RRID:AB_2616028.

Peak calling parameters

The GRCz11 index was built using Burrows–Wheeler Aligner (BWA, v0.7.12), and clean reads were then mapped to the reference genome using BWA-MEM (v0.7.12). The MACS2 (version 2.1.0) peak calling software to identify regions of IP enrichment over background. A q-value threshold of 0.05 was used for all data sets. After peak calling, the distribution of chromosome distribution, peak width, fold enrichment, significant level and peak summit number per peak were all displayed.

Data quality

Raw data (raw reads) of fastq format were firstly processed using fastp software. In this step, clean data (clean reads) were obtained by removing reads containing adapter, reads containing ploy-N and low quality reads from raw data. At the same time, Q20, Q30 and GC content of the clean data were calculated. All the downstream analyses were based on the clean data with high quality.

Software

fastp (version 0.19.11);
BWA (v 0.7.12);
BWA (v 0.7.12);
MACS2 (version 2.1.0).
